# Covariate-constrained randomization in cluster randomized 2x2 factorial trials: Application to a diabetes prevention study

**DOI:** 10.21203/rs.3.rs-3783684/v1

**Published:** 2024-03-25

**Authors:** Juned Siddique, Zhehui Li, Matthew J. O’Brien

**Affiliations:** 1 Department of Preventive Medicine, Northwestern University Feinberg School of Medicine, 680 N Lake Shore Drive, Suite 1400, Chicago, IL, USA; 2 Department of Medicine, Northwestern University Feinberg School of Medicine, 750 N Lake Shore Drive, 10th floor, Chicago, IL, USA

**Keywords:** CRT, balance, confounding

## Abstract

**Background:**

Cluster randomized trials (CRTs) are randomized trials where randomization takes place at an administrative level (e.g., hospitals, clinics, or schools) rather than at the individual level. When the number of available clusters is small, researchers may not be able to rely on simple randomization to achieve balance on cluster-level covariates across treatment conditions. If these cluster-level covariates are predictive of the outcome, covariate imbalance may distort treatment effects, threaten internal validity, lead to a loss of power, and increase the variability of treatment effects. Covariate-constrained randomization (CR) is a randomization strategy designed to reduce the risk of imbalance in cluster-level covariates when performing a CRT. Existing methods for CR have been developed and evaluated for two- and multi-arm CRTs but not for factorial CRTs.

**Methods:**

Motivated by the BEGIN study—a CRT for weight loss among patients with pre-diabetes—we develop methods for performing CR in 2x2 factorial cluster randomized trials. We apply our methods to the BEGIN study and use simulation to assess the performance of CR versus simple randomization for estimating treatment effects by varying the number of clusters, the degree to which clusters are associated with the outcome, the distribution of cluster level covariates, and analysis strategies.

**Results:**

Compared to simple randomization of clusters, CR in the factorial setting is effective at achieving balance across cluster-level covariates between treatment conditions and provides more precise inferences. When cluster-level covariates are included in the analyses model, CR also results in greater power to detect treatment effects, but power is low compared to unadjusted analyses when the number of clusters is small.

**Conclusions:**

CR should be used instead of simple randomization when performing factorial CRTs to avoid highly imbalanced designs and to obtain more precise inferences. Except when there are a small number of clusters, cluster-level covariates should be included in the analysis model to increase power and maintain coverage and Type 1 error rates at their nominal levels.

## 1 Background

Cluster randomized trials (CRTs) are randomized controlled trials where randomization takes place at an administrative level (e.g., hospitals, clinics, or schools) rather than at the individual level. CRTs are an attractive research design when there are concerns of treatment contamination among participants, when it is logistically easier to conduct the trial by randomizing at the cluster level, and when the intervention of interest is delivered at the cluster level [1].

A major practical limitation when conducting CRTs is the ability to enroll a large number of clusters. When the number of available clusters is small, researchers may not be able to rely on simple randomization to achieve balance on cluster-level covariates across treatment conditions [2]. If these cluster-level covariates are predictive of the outcome, covariate imbalance across treatment conditions may distort treatment effects, threaten internal validity, lead to a loss of power, increase the variability of the treatment effect, and usually requires statistical adjustment in the analysis stage [3]. For example, in a two-arm CRT where clinics are randomized to treatment conditions and where the size of a clinic is related to the outcome of interest, researchers would want equal numbers of small and large clinics in the treatment and control conditions, respectively.

Factorial experiments are an efficient approach to determine which of several possible components of a proposed intervention have effects of practical significance [4]. When implementing factorial experiments at the cluster level, the challenges involved in balancing cluster-level covariates across arms is magnified because there are more than two treatment conditions. For example, in a 2x2 factorial CRT, clusters will be randomized to one of 4 treatment conditions.

One approach to address imbalance in prognostic cluster-level covariates across treatment conditions is to include these covariates in the analysis model which can help ensure an unbiased estimate of the treatment effect. The drawback to including cluster-level covariates in the analysis model is the subsequent loss of degrees of freedom that are available to estimate treatment effects. This resulting loss of power can be substantial when there are a small number of clusters [5]. An alternative to model-based covariate adjustment is to control for potential confounders at the *design* stage, by balancing the distribution of select measured characteristics across treatment arms. This can help ensure more precise treatment effects as well as confidence that observed treatment effects are not due to imbalance in prognostic covariates while at the same time avoiding the resulting loss of power due to covariate adjustment.

Individually randomized trials often rely on stratification to achieve balance on prognostic factors across treatment conditions. In CRTs with a small number of clusters, stratifying on more than one variable can be challenging because of an insufficient number of clusters to distribute among strata. This phenomenon is only exacerbated in factorial trials where there are at least 4 treatment conditions. For example, with two binary stratification variables there will be total of four strata. To conduct a 2x2 factorial CRT would require at least 4 clusters per stratum (16 clusters total) to avoid unequal allocation of treatments within strata [3]. Furthermore, stratifying on a continuous factor requires converting it to a categorical variable, a process that can result in a loss of information.

Covariate-constrained randomization (CR) is an alternative procedure for achieving balance across treatment conditions on a set of pre-specified cluster-level covariates. Unlike individual level trials where participants are recruited sequentially, the participating units in a CRT are generally assembled at the start of the study so that cluster-level covariate values such as geographic location, clinic size, and the income level of patients are available at the design stage.

The first step in CR is to identify those cluster-level covariates that are predictive of the outcome on which one wishes to achieve balance. Using the terminology of Li et al. [6], we refer to these covariates as “potential confounders” because they are cluster-level prognostic factors that, when imbalanced, could distort estimates of treatment effects.

The second step in CR is—for every possible randomization scheme (or a random subset of schemes when the number of clusters is large)—to calculate a balance score that measures the difference in the distribution of cluster-level covariates across treatment conditions [3, 7]. Next, a subset of schemes is chosen that meet some pre-specified balance criteria, such the 10% of schemes with the best balance scores. Finally, an allocation is randomly selected among those schemes that meet the pre-specified criteria and is used to randomize clusters. CR tends to produce better balance on average across treatment conditions as compared to simple randomization in which a randomization scheme is selected from all possible schemes with equal probability assigned to each scheme. Compared with stratification, CR may be preferred due to its capacity to accommodate multiple covariates, both categorical and continuous [8].

There are numerous variations of CR that use different balance metrics and different analysis strategies. In the two-arm setting, Raab and Butcher [7] and Li et al. [6] consider weighted and unweighted pairwise balance scores based on the difference in covariate means between arms. In the multi-arm setting, Zhou et al. [9] extend the pairwise balance score method, while Watson et al. [10] present a balance metric based on the sum of cluster-level mean differences. Ciolino et al. [11] calculate a Kruskal-Wallis test for each covariate across arms and assesses balance based on the p-values of these tests where a minimum p-value greater than 0.30 was found to appropriately identify acceptable balance.

Li et al. [6], Watson et al. [10], and Zhou et al. [9] also recommend adjustment for potential confounders in the analyses stage to maintain Type 1 error and provide adequate power. The most common approach for the analysis of CRTs is mixed-effects regression modeling with random cluster-level effects to account for within-cluster correlation. Mixed-effects models are sufficiently flexible to allow for adjustment of both cluster-level and participant-level covariates. Random effects at the participant level can be included if, for example, the study has repeated observations on the same individuals.

Existing work on CR has focused on two- or three-arm CRTs. The performance of CR in a factorial setting—where the minimum number of randomization conditions is 4—has not been explored. Unlike in multi-arm trials, in CRT factorial designs, clusters are “recycled” [4] when estimating treatment effects so that every cluster is used in every estimate of a treatment effect. Whether CR operates differently in this setting is an area that requires further investigation.

### 1.1 Motivating Example

Our methods are motivated by the Behavioral Nudges for Diabetes Prevention (BEGIN) study [12], a 2x2 factorial CRT studying two pragmatic behavioral interventions that prompt patients to adopt evidence-based treatment for prediabetes in primary care, thereby promoting modest weight loss. Preventing Type 2 diabetes (T2D) has become a top public health priority given the high prevalence of prediabetes and the availability of evidence-based treatments to prevent T2D [13, 14]. With 682 million office visits made by U.S. adults annually, primary care is a critical venue for promoting weight loss and T2D prevention [15].

BEGIN takes place at the Erie Family Health Center, a Federally-funded primary care clinic network in Chicago serving 85,000 vulnerable patients, 83% of whom live in poverty and 79% of whom are Hispanic/Latino. Given their reach and unique access to high-risk populations, community health centers are an ideal venue for studying primary care-based interventions that promote prediabetes treatment uptake and modest weight loss.

The two BEGIN primary care interventions are: 1) in-person behavioral nudges via a prediabetes decision aid delivered by existing health educators; and 2) automated behavioral nudges via motivational letters and text messages. These two interventions are being tested in 8 Erie Family Health Center clinics using a 2x2 factorial design. Two clinics are randomly assigned to each of the the four conditions in Table 1. These four conditions are:

- In-person intervention alone
- Text message intervention alone
- Both in-person and text message interventions
- Neither intervention

Because randomization occurs at the clinic level, there is a risk of imbalance in clinic-level characteristics across treatment conditions. Table 2 presents data on three clinic-level covariates from the 8 clinics in the BEGIN study on which the BEGIN investigators sought to achieve balance. The data in Table 2 are based on clinic visits in 2019–2020 (prior to the start of the BEGIN study) among patients who met the eligibility criteria of the BEGIN study. These three potential confounders are: 1) Clinic volume, as measured by the number of office visits; 2) Percent of office visits by female patients; 3) Mean BMI of visits. It is worth noting that mean BMI is similar across the 8 clinics, but total volume varies considerably.

In this manuscript, motivated by the BEGIN study, we extend and evaluate CR methods for multi-arm trials [6, 7, 10] to the 2x2 factorial CRT setting. The outline for the rest of this paper is as follows. In Section 2, we present methods for CR in the setting of a 2x2 factorial CRT and describe a simulation study to assess the performance of our methods as compared to simple randomization of clusters. In Section 3, we present the results of our simulation study and apply our methods to the BEGIN study. Section 4 provides discussion and areas of future work. We conclude in Section 5.

## 2 Methods

As mentioned above, once a set of potential cluster-level confounders are identified, the next step in performing CR is to calculate a balance metric to measure the difference in the distribution of these cluster-level covariates across treatment conditions for all possible randomization schemes. In this section we describe a balance metric for factorial trials that extends the balance metrics of Li et al. [6], Raab and Butcher [7] and Watson et al. [10].

Let J be the number of clusters and T be the number of treatment conditions so that $n_{T}=\frac{J}{T}$ clusters are randomized to each treatment condition. Let $x_{jk}$ be the value of the *k*^*th*^ covariate (k=1,…,K) in cluster j(j=1,…,J), and ${\overset{‾}{x}}_{tk}=\frac{1}{n_{T}}\sum_{j\in t}\;x_{jk}$ the mean value of the *k*^*th*^ covariate in clusters assigned to condition t,(t=1,…,T). Finally ${\overset{‾}{x}}_{k}=\frac{1}{J}\sum_{j=1}^{J}\;{\overset{‾}{x}}_{jk}$ is the overall mean of covariate k across all clusters. Our balance metric is:

(1) $$B=\sum_{k=1}^{K}w_{k}\sum_{t=1}^{T}{\left({\overset{-}{x}}_{tk}-{\overset{-}{x}}_{k}\right)}^{2}$$

where $w_{k}$ is a predetermined weight for the *k*th covariate. Following Raab and Butcher [7] and Li et al. [6], we set $w_{k}$ as the inverse of the variance of the *k*th covariate across all clusters. That is

(2) $$w_{k}=\frac{1}{s_{k}^{2}}=\frac{n\;-\;1}{\sum_{j=1}^{J}\;\;{\left(x_{jk}\;-\;{\overset{‾}{x}}_{k}\right)}^{2}}.$$

The metric in (1) and (2) describe the balance score introduced by Watson et al. [10] for use in multi-arm trials. A limitation to this metric is that balance is purely defined by covariate values and does not take into account clinical importance. For example, in the BEGIN study, if clinic volume is considered to be a stronger predictor of weight loss than percent of female visits, we may want to give clinic volume greater weight in the balance metric so that smaller balance scores using the weighted metric will reflect better balance on clinic volume at the expense of less balance on clinic percent female. To incorporate weights into the balance metric in (1) we use the approach of Yu et al. [8] to produce the weighted balance metric:

(3) $$B_{w}=\sum_{k=1}^{K}d_{k}w_{k}\sum_{t=1}^{T}{\left({\overset{-}{x}}_{tk}-{\overset{-}{x}}_{\cdot k}\right)}^{2}$$

where $d_{k}$ is a user-defined weight for the *k*th covariate. When $d_{k}=1$ for all covariates, then (3) reduces to the balance metric in (1). When researchers consider certain variables to be more predictive of the outcome than others or for which there is greater variability across clusters, a user-defined weight $d_{k}>1$ could be assigned to those variables when calculating balance scores [6].

To perform CR, the balance metric B (or $B_{w}$) is generated for all possible randomization schemes of the J clusters. The final allocation is chosen from a subset of allocations that meet a pre-specified balance criteria. Here, we select a cutoff value q which is the q th percentile of the balance scores. Yu et al. [8] note that the cutoff value q should be small and away from 1.0 (simple randomization) so that only the more balanced randomization schemes are retained in the constrained space. Following Yu et al. [8], we set q=0.1 so that only the schemes in the top 10% of balance scores are included in our constrained allocation space.

When the number of clusters is small, it is feasible to calculate the balance score for all possible allocations where the number of allocations is $\frac{J!}{{\left[(J/T)!\right]}^{T}}$. For example, when J=8 and T=4, there are only 2520 possible ways to randomize clusters. However, for CRTs with more clusters, for example, when J=12 and T=4, there are 369,600 possible ways to randomize the clusters and enumerating all possible allocations becomes computationally expensive. Following Li et al. [6], when J>8, we randomly sample a subset of 20,000 allocations from all possible allocations, then select our final allocation from the top 10% (2,000) of allocations in terms of balance scores.

### 2.1 Simulation Study

We use simulation to assess our method of CR in the setting of a 2x2 factorial cluster randomized trial and how it compares to simple randomization in terms of estimating treatment effects. Following Li et al. [6] we simulate data using the following approach. Let $x_{j1}$, $x_{j2}$, $x_{j3}$ be three independent cluster level covariates for cluster j, (j=1,…,J); that are normally distributed with mean 1 and variance $\sigma_{x}^{2}$ on which we wish to achieve balance. Let $y_{ij}$ be the outcome of interest for subject $i\;\left(i=1,\ldots ,n_{j}\right)$; in cluster j. We set $n_{j}=100$ throughout. Let ${Trt}_{1j}$ and ${Trt}_{2j}$ indicate whether cluster j is assigned to treatments 1 and/or 2, respectively, where treatment is based on the factorial design in Table 1. We generate $y_{ij}$ from the following linear mixed-effects model:

(4) $$y_{ij}=\beta_{1}x_{j1}+\beta_{2}x_{j2}+\beta_{3}x_{j3}+\gamma_{1}{Trt}_{1j}+\gamma_{2}{Trt}_{2j}+b_{0j}+\varepsilon_{ij}$$

The parameters $\beta_{1}$, $\beta_{2}$, and $\beta_{3}$ are regression coefficients on the cluster-level covariates that are predictive of the outcome (when β≠0). For simplicity, we let $\beta_{1}=\beta_{2}=\beta_{3}$. The coefficients $\gamma_{1}$ and $\gamma_{2}$ correspond to the effects of the two interventions. We set $\gamma_{1}=5$ and $\gamma_{2}=0$. The parameter $b_{0j}$ is a cluster-level random effect where $b_{0j}\sim N\left(0,\;\sigma_{b}^{2}\right)$ and $\varepsilon_{ij}$ is an error term where $\varepsilon_{ij}\sim N\left(0,\sigma_{\varepsilon }^{2}\right)$. We assume $\sigma_{\varepsilon }^{2}=36$ and an intra-cluster correlation (ICC) of ρ=0.05 so that $\sigma_{b}^{2}=\rho \sigma_{e}^{2}/(1-\rho )$.

We sought to investigate the following factors in our simulation study and examine how their effects differ when using CR as compared to simple randomization: number of clusters, the variability of cluster-level covariates, the magnitude of cluster-level effects on the outcome, and whether or not cluster-level covariates are controlled for in the analysis model. Table 3 shows the factors that vary in the simulation. With five factors with two or three levels each, we evaluated a total of 2 × 2 × 3 × 3 × 2 = 72 scenarios. Simulation is based on the following steps:

1. Simulate K=3 independent cluster level covariates of size J, where $x_{jk}\sim N\left(1,\sigma_{x}^{2}\right)$.
2. Use either CR (see code in Appendix A for implementing CR in R) or simple randomization to randomize the J clusters to one of the 4 conditions in Table 1.
3. Draw $\varepsilon_{ij}\sim N\left(0,\sigma_{e}^{2}\right)$, $i=1,\ldots ,n_{j}$; j=1,…,J. Here we fix $\sigma_{e}^{2}=36$.
4. Draw $b_{0j}\sim N\left(0,\sigma_{b}\right)$, j=1,…,J where $\sigma_{b}=\rho \sigma_{e}^{2}/(1-\rho )$, and ρ=0.05 is the ICC.
5. Generate $n_{j}=100$ values of $y_{ij}$ using (4).
6. Analyze the data using a linear mixed-effects model with a random intercept for cluster and indicator variables for the two treatment conditions. Based on the simulation scenario, the analysis model either controls for or does not control for cluster-level covariates.

When controlling for cluster-level covariates in the analysis model, the analysis model is identical to (4). When the analysis model does not control for cluster-level covariates, the analysis model excludes $x_{j1}$, $x_{j2}$, $x_{j3}$.

Steps 1–6 were performed 10,000 times to generate 10,000 parameter estimates for each of the 72 simulation scenarios. We focus our attention on the performance of the treatment effects γ. Specifically, using $\gamma_{1}$ we assess the percent bias, variance, mean squared error (MSE), coverage and width of the 95% confidence interval, and the power to reject the null hypothesis. Using $\gamma_{2}$, we assess Type 1 error.

## 3 Results

### 3.1 Simulation Results

Tables 4 and 5 summarize the results of our simulation study for 8 and 12 clinics, respectively, using both CR and simple randomization under various degrees of cluster-level variability $\left(\sigma_{x}\right)$ and cluster-level covariate effects (β). The results in Tables 4 and 5 are from simulations where cluster-level covariates are not controlled for in the analysis model.

Looking at Table 4, across all scenarios, the percent bias is essentially 0 for both CR and simple randomization. As the magnitude of cluster-level covariate effects increases (as measured by β) variance and MSE increase, with both performance criteria better under CR. A similar trend is seen with increasing values of cluster-level variability (as measured by $\sigma_{x}$), where variance and MSE increase as $\sigma_{x}$ increases and both performance criteria are lower under CR. Coverage and Type 1 error tend to be conservative under CR while these values are at their nominal levels under simple randomization.

Power in Table 4 is similar for both CR and simple randomization. However, in those settings where the magnitude of potential confounding is high and cluster-level variability is also high, power is low for both CR and simple randomization. For example, when $\sigma_{x}=2$ and β=1, power is 24% under CR and 33% under simple randomization. As expected, covariate balance is better under CR compared to simple randomization. Because the balance metric in (1) standardizes each covariate by the inverse of its variance, values of $\sigma_{x}$ do not have an effect on the balance metric and balance is the same across all values of $\sigma_{x}$ under CR.

The results in Table 5 based on 12 clusters are similar to those based on 8 clinics, with better variance and MSE under CR and similar power as compared to simple randomization. Again, coverage and Type 1 error are conservative under CR while these criteria are at their nominal level under simple randomization. However, with 12 clusters, power is much greater than in the setting with 8 clusters such that power is only inadequate in the scenario with the highest potential confounding (β=1) and the highest between-cluster variability $\left(\sigma_{x}=2\right)$.

It is also worth noting that in Tables 4 and 5, the performance criteria are the same when the *product* of $\sigma_{x}$ and β are the same. For example, the performance criteria when $\sigma_{x}=0.5$ and β=1.0 are the same as when $\sigma_{x}=1.0$ and β=0.5. This is because while the *conditional* variance of the outcome is the same in all the simulation scenarios, in the unadjusted analyses, we do not condition on the cluster-level covariates so that the variance of the outcome varies across all simulation scenarios and is reflected in an inflated between-cluster variance. That is

(5) $$Var\left(y_{ij}\right)\;=E\left\{Var\left(y_{ij}|x_{j}\right)\right\}+Var\left\{E\left(y_{ij}|x_{j}\right)\right\}\;\;=\sigma_{e}^{2}+\sigma_{b}^{2}+3\beta^{2}\sigma_{x}^{2}$$

where the term $3\beta^{2}\sigma_{x}^{2}$ is the increase in variance due to not conditioning on covariates. When the analysis model does condition on covariates, $Var\left(y_{ij}|x_{j}\right)=\sigma_{e}^{2}+\sigma_{b}^{2}$.

Appendix Tables 7 and 8 summarize the simulation results for 8 and 12 clusters, respectively, now based on an analysis model that controls for cluster-level covariates. Here, the analysis model is identical to the data generating model so that the results for CR are the same across all scenarios and the results for simple randomization are the same across all scenarios. Overall, even when controlling for covariates in the analyses, there is a benefit to using CR as compared to simple randomization in terms of lower MSE, greater power, and narrower confidence interval width. And unlike in the unadjusted analyses, coverage and Type 1 error are not conservative and are at their nominal levels when using CR with 12 clusters.

Comparing simulations with 8 clusters where the analyses does not control for covariates (Table 4) to simulations with 8 clusters where the analysis model does control for covariates (Table 7) we see that controlling for covariates has an especially adverse effect on power such that power is only 54% under CR and 43% under simple randomization. The only scenario where controlling for covariates produces better results than not controlling for covariates is the extreme scenario with the highest potential confounding and the highest between-cluster variability. Here, variance, MSE, power, and CI width are all better when controlling for covariates.

With 12 clusters (Table 8) there appears to be a clear advantage to controlling for cluster-level covariates in the analyses. The effect on power as compared to not controlling for covariates (Table 5) is modest, and in those scenarios with a high degree of potential confounding, controlling for covariates results in a marked increase in power. For example, under CR in the scenario with the highest potential confounding and the highest between-cluster variability, power goes from 0.49 when not controlling for covariates to 0.98 when controlling for covariates. And as mentioned earlier, coverage and Type 1 error are at their nomial levels when controlling for covariates.

### 3.2 Application to the BEGIN study

**Figure 1:**
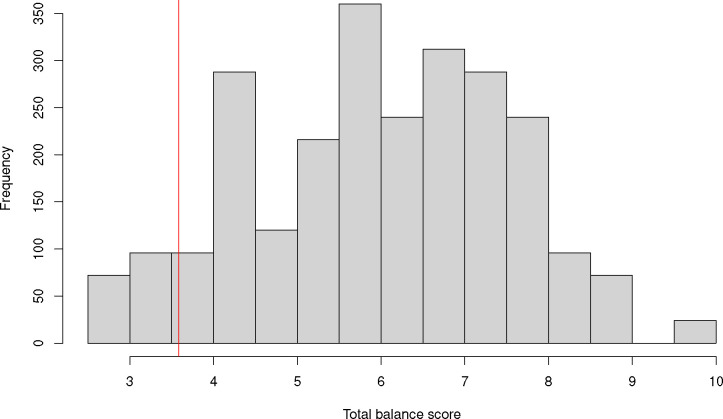
Histogram of total balance scores for the 2520 possible allocation schemes for the BEGIN cluster randomized trial with 8 clusters and 4 randomization conditions. The vertical red line indicates the cutoff corresponding to the top 10% of balance scores among the 2520 possible scores.

We applied our methods for CR in factorial trials to the BEGIN study, using the cluster-level covariate information in Table 2. With 8 clusters and 4 treatment conditions there are $\frac{8!}{2^{4}}=2520$ possible schemes. Using the balance metric in (3), we calculated the balance score for each of these possible 2520 allocation schemes. Based on a belief by the BEGIN investigators that clinic volume was an important predictor of weight loss, and the fact that mean BMI was similar across all clinics, clinic volume was given a weight of 2 in (3), while percent female and mean BMI were given weights of 1. Figure 1 displays a histogram of the balance scores for all 2520 possible schemes. The vertical red line in Figure 1 indicates the cutoff corresponding to the top 10% balance scores among the 2520 scores.

In our setting, for a given set of clinic matches, the treatment assignments can be labeled 4! = 24 different ways, so that our 2520 possible allocations correspond to only 2520/24 = 105 unique balance scores. The allocations corresponding to the top 10 unique balance scores are listed in Table 6.

Note that in seven of the ten allocations in Table 6, clinics C7 and C8 are matched together. Clinics C7 and C8 have the largest and smallest clinic volumes, respectively. Assigning them to the same treatment condition helps ensure balance across treatment conditions. Conversely, clinics C1 and C2 are only matched together in two of the ten allocations. Clinics C1 and C2 are the second and third largest clinics. Putting them in *different* treatment conditions also helps ensure balance.

## 4 Discussion

In this paper we presented a method for performing CR in factorial cluster randomized trials. We performed a simulation study to assess the effectiveness of our method as compared to simple randomization in terms of estimating treatment effects in the setting of a 2x2 factorial trial. In all scenarios, bias of the treatment effect was essentially 0. However, by balancing prognostic covariates across treatment arms, CR resulted in more precise estimates of the treatment effect as measured by MSE, a finding also noted by Kalish and Begg [16]. And by constraining the allocation space, CR eliminates the possibility of a highly imbalanced allocation which may significantly undermine the power of a trial as well as threaten its internal validity [10]. When covariates were not controlled for in the analysis model, we found that both CR and simple randomization produced similar rates of power but coverage and Type 1 error rates were conservative under CR, a finding that was also found in Li et al. [6].

When covariates were controlled for in the analysis, simulations again showed a clear benefit of CR versus simple randomization across all performance criteria in addition to coverage and Type 1 error close to or at their nominal levels. Still, the question of whether or not one should control for covariates in the analysis model is not clear-cut. The rationale to control for cluster-level covariates even when performing CR is that including these covariates helps adjust for any residual imbalances not controlled for during randomization and can also reduce residual variance. The trade-off is a reduction in the number of degrees of freedom for estimating treatment effects. For example, when there are 8 clusters and covariates are not included in the analysis model, there are 8 − 3 = 5 degrees of freedom available to estimate the treatment effects. Including 3 cluster-level covariates in the analysis model reduces this to only 2 degrees of freedom.

In our simulations with 8 clusters, the loss of power when controlling for covariates was so substantial that controlling for covariates is not recommended due to the decrease in degrees of freedom for estimating treatment effects. This loss of power highlights another benefit of CR—it allows to user to control for cluster-level covariates in order to avoid highly imbalanced designs and obtain more precise inferences—without the resulting decrease in degrees of freedom that would occur if covariates were controlled for in the analysis model. With 12 clusters, the loss of power when controlling for covariates in the analysis model was minimal, and in some scenarios produced better power than not controlling for covariates.

When cluster-level covariates have small variance, as was the case in our simulations when $\sigma_{x}=0.5$, there is little benefit to controlling for covariates in the analysis model and a substantial loss of power. This can be seen by comparing Table 4 and Appendix Table 7 when $\sigma_{x}=0.5$ and β=1. Here, power is 89% when not controlling for covariates but only 54% when covariates are included in the analysis model. Only when $\sigma_{x}=2$ and the degree of confounding is high is power better when controlling for covariates.

This finding is relevant to the BEGIN study, where there are only 8 clusters and the variability in the cluster-level covariates is small. In our simulation studies, where the mean of the covariates was 1, the coefficient of variation in the cluster-level covariates ranged from 0.7 when $\sigma_{x}=0.5$, to 1.4, when $\sigma_{x}=2$. In Table 2, the clinic volume coefficient of variation is 0.44. But the coefficient of variation for percent female is 0.08 and the coefficient of variation for mean BMI is only 0.01. These values suggest that the analysis model for the BEGIN study should not control for clinic-level covariates unless the distribution of clinic-level covariates in the actual trial data is much different from the values in Table 2.

There are several limitations to our study. CR requires the enumeration of all possible allocations. In a 2x2 factorial study, it is only feasible to enumerate all possible allocations when there are 8 clusters. When we simulated data with 12 clusters, we randomly sampled 20,000 allocations following the approach in Li et al. [6]. We did not investigate whether this sample size is large enough to adequately represent all possible allocations. Furthermore, we did not assess the size of our constrained allocation space and used the top 10% of balanced allocations throughout. As shown by Li et al. [6], an overly constrained allocation space can result in conservative Type 1 error rates which was the case in our unadjusted analyses. Expanding the allocation space may reduce this phenomenon while still retaining the benefits of CR over simple randomization. Finally, we evaluated our balance metric using simulated continuous covariates. Future work will evaluate how well our methods perform when binary or categorical group-level covariates are used to constrain the randomization set.

## 5 Conclusions

Our findings provide evidence for the use of CR instead of simple randomization when performing factorial CRTs to avoid highly imbalanced designs and to obtain more precise inferences. Except when there are a small number of clusters per treatment condition, cluster-level covariates should be included in the analysis model to increase power and produce coverage and Type 1 error rates at their nominal levels. When there are a small number of clusters, we recommend cluster-level covariates should not be included in the analysis model due to the loss of power even though coverage and Type 1 error rates will be conservative in the unadjusted analyses.

## Figures and Tables

**Table 1:** 2x2 factorial design of the BEGIN Study

	Intervention	
Condition	In-person Text messages	

a	on	off
b	off	on
c	on	on
d	off	off

**Table 2:** Total volume, percent female, and mean BMI of visits by patients who met the BEGIN eligibility criteria in 2019–2020 for each of the 8 clinics in the BEGIN trial.

Clinic Number	Total Volume	Percent Female	Mean BMI

C1	29933	73.57	31.19
C2	26613	88.54	31.20
C3	23940	77.59	31.53
C4	18869	77.52	30.55
C5	14660	84.65	30.32
C6	24119	81.71	31.11
C7	34637	74.39	30.58
C8	3429	71.19	31.33

**Table 3:** Factors that vary in the simulation study

Factor	Values

Number of clusters *J*	*J* = 8, 12
Randomization type	Simple, Covariate-constrained
SD of Cluster-level covariates *σ_x_*	0.5, 1, 2
Cluster-level covariate effects *β*	None (*β* = 0), Medium (*β* = 0.5), Large (*β* = 1)
Analysis model	Control/Do not control for covariates

**Table 4:** Simulation results for the effect of treatment with 8 clusters, based on an analysis model that does not control for cluster-level covariates

Covariate SD	Degree Confounding	%Bias	Var	MSE	Cov	Power	CI Width	Type 1 Error	Balance

*Covariate Constrained Randomization*									
	*β* = 0.0	−0.17	1.15	1.15	0.95	0.96	5.18	0.05	2.19
*σ_x_* = 0.5	*β* = 0.5	−0.17	1.21	1.21	0.95	0.94	5.44	0.05	2.19
	*β* = 1.0	−0.17	1.35	1.35	0.96	0.89	6.15	0.04	2.19

	*β* = 0.0	−0.18	1.15	1.15	0.95	0.96	5.18	0.05	2.19
*σ_x_* = 1	*β* = 0.5	−0.17	1.35	1.35	0.96	0.89	6.15	0.04	2.19
	*β* = 1.0	−0.17	1.91	1.91	0.98	0.67	8.40	0.02	2.19

	*β* = 0.0	−0.17	1.15	1.15	0.95	0.96	5.18	0.05	2.19
*σ_x_* = 2	*β* = 0.5	−0.17	1.91	1.91	0.98	0.67	8.40	0.02	2.19
	*β* = 1.0	−0.16	4.13	4.13	0.99	0.24	14.21	0.01	2.19

*Simple Randomization*									
	*β* = 0.0	−0.06	1.17	1.17	0.95	0.96	5.17	0.05	4.50
*σ_x_* = 0.5	*β* = 0.5	0.00	1.25	1.25	0.95	0.94	5.39	0.05	4.50
	*β* = 1.0	0.06	1.51	1.51	0.95	0.89	5.99	0.05	4.50

	*β* = 0.0	−0.06	1.17	1.17	0.95	0.96	5.17	0.05	4.50
*σ_x_* =1	*β* = 0.5	0.06	1.51	1.51	0.95	0.89	5.99	0.05	4.50
	*β* = 1.0	0.18	2.61	2.61	0.95	0.70	7.95	0.05	4.50

	*β* = 0.0	−0.06	1.17	1.17	0.95	0.96	5.17	0.05	4.50
*σ_x_* = 2	*β* = 0.5	0.18	2.61	2.61	0.95	0.70	7.95	0.05	4.50
	*β* = 1.0	0.41	7.03	7.03	0.95	0.33	13.10	0.05	4.50

**Table 5:** Simulation results for the effect of treatment with 12 clusters, based on an analysis model that does not control for cluster-level covariates

Covariate SD	Degree Confounding	%Bias	Var	MSE	Cov	Power	CI Width	Type 1 Error	Balance

*Covariate Constrained Randomization*									
	*β* = 0.0	−0.44	0.76	0.76	0.95	1.00	3.81	0.05	1.27
*σ_x_* = 0.5	*β* = 0.5	−0.51	0.78	0.79	0.95	1.00	3.98	0.04	1.27
	*β* = 1.0	−0.57	0.86	0.86	0.96	0.99	4.46	0.03	1.27

	*β* = 0.0	−0.45	0.76	0.76	0.95	1.00	3.81	0.05	1.27
*σ_x_* = 1	*β* = 0.5	−0.57	0.86	0.86	0.96	0.99	4.46	0.03	1.27
	*β* = 1.0	−0.70	1.18	1.18	0.98	0.93	6.03	0.02	1.27

	*β* = 0.0	−0.44	0.76	0.76	0.95	1.00	3.81	0.05	1.27
*σ_x_* = 2	*β* = 0.5	−0.71	1.18	1.18	0.98	0.93	6.03	0.02	1.27
	*β* = 1.0	−0.97	2.44	2.44	0.99	0.49	10.11	0.01	1.27

*Simple Randomization*									
	*β* = 0.0	−0.02	0.75	0.75	0.95	1.00	3.80	0.05	2.98
*σ_x_* = 0.5	*β* = 0.5	−0.06	0.81	0.81	0.95	1.00	3.96	0.05	2.98
	*β* = 1.0	−0.09	0.99	0.99	0.95	0.99	4.40	0.05	2.98

	*β* = 0.0	−0.02	0.75	0.75	0.95	1.00	3.80	0.05	2.98
*σ_x_* = 1	*β* = 0.5	−0.09	0.99	0.99	0.95	0.99	4.40	0.05	2.98
	*β* = 1.0	−0.16	1.72	1.72	0.95	0.92	5.83	0.05	2.98

	*β* = 0.0	−0.02	0.75	0.75	0.95	1.00	3.80	0.05	2.98
*σ_x_* = 2	*β* = 0.5	−0.16	1.72	1.72	0.95	0.92	5.83	0.05	2.98
	*β* = 1.0	−0.30	4.70	4.70	0.95	0.53	9.60	0.05	2.98

**Table 6:** Clinic pairings associated with the top 10 unique balance scores sorted by total balance score, using data from the BEGIN study.

				Clinic						Balance Score		
Allocation	C1	C2	C3	C4	C5	C6	C7	C8	Total	Female	Volume	BMI

1	a	b	c	b	a	c	d	d	2.79	1.56	0.29	0.94
2	a	b	c	b	c	a	d	d	2.85	1.73	0.89	0.23
3	a	a	b	c	b	c	d	d	2.92	1.35	1.18	0.39
4	a	b	c	c	a	d	d	b	3.10	0.09	2.19	0.82
5	a	b	c	c	a	b	d	d	3.11	2.22	0.44	0.45
6	a	b	b	c	a	c	d	d	3.17	1.57	0.42	1.18
7	a	b	c	d	a	d	c	b	3.29	0.28	2.16	0.85
8	a	b	c	a	c	d	d	b	3.57	0.50	2.46	0.61
9	a	b	c	a	c	b	d	d	3.58	2.63	0.70	0.25
10	a	a	b	b	c	c	d	d	3.58	1.77	1.17	0.65

## Appendix A R code for covariate-constrained randomization in the BEGIN 2x2 factorial trial

The R code below is to implement covariate constrained randomization in the BEGIN 2x2 factorial trial. The code below is for three potential cluster-level confounders and 4 randomization conditions.

CR_fn <- function(data, x1.d=1, x2.d=1, x3.d=1, nsample=20000){ 
  # function to perform covariate-constrained randomization 
  # Args:
  # data: dataframe of 3 cluster-level covariates (x1, x2, x3)
  # x1.d, x2.d, x3.d: user-defined weights
  # nsample: number of sampled allocations to be used
  # when there are more than 8 clusters
  #
  # Returns:
  # The data dataset with treatment assignment and total balance appended
  # required packages 
  library(doBy) 
  library(arrangements)
  # If 8 clinics then enumerate all possible permutations 
  if(nrow(data) == 8) nsample <- NULL else nsample <- nsample
  data <- as.data.frame(data) 
  J <- nrow(data)
  # calculate inverse of variance 
  x1.w <- 1/var(data$x1) 
  x2.w <- 1/var(data$x2) 
  x3.w <- 1/var(data$x3)
  # calculate the balance scores
  # generate permutations for J clinics and 4 conditions 
  perms <- permutations(4, freq=c(J/4, J/4, J/4, J/4), nsample=nsample)
  B <- rep(0, nrow(perms)*4) # vector of balance scores 
  dim(B) <- c(nrow(perms), 4) 
  for(i in 1:nrow(perms)){
    data$tx <- perms[i,] # calculate by permutation 
    means <- summaryBy(cbind(x1, x2, x3) ~ tx, data=data) 
  # balance score is the sum of squared deviations of the covariate
  # means multiplied by their weight which is the inverse of the cluster variances 
  x1.bal <- (nrow(means)-1) * var(means$x1.mean) * x1.w * x1.d 
  x2.bal <- (nrow(means)-1) * var(means$x2.mean) * x2.w * x2.d 
  x3.bal <- (nrow(means)-1) * var(means$x3.mean) * x3.w * x3.d
  # add up covariate-specific balance scores 
  total.bal <- sum(x1.bal, x2.bal, x3.bal)
  #return all balance scores
  B[i,1:4] <- c(total.bal, x1.bal, x2.bal, x3.bal)
}
  # merge balance scores with permutations data set
  permdata <- data.frame(perms) 
  permdata <- cbind(permdata, B)
  names(permdata) <- c(“1”:J, “total.bal”, “x1.bal”, “x2.bal”, “x3.bal”)
  # randomly choose one allocation from the top 10% balance score
  # sort by total balance score
  permsort <- permdata[order(permdata$total.bal),]
  # Now random select from top 10% of balance scores
  n <- ifelse(J==8, (factorial(J)/factorial(J/4)^4)*0.1, 0.1*nsample)
  ralloc <- sample(1:n, 1)
  CR.trt <- permsort[ralloc,]
  # assign treatment 1 (Decision Support) to those clinics who were
  # randomized to conditions 1 or 3
  trt1 <- as.numeric(CR.trt[,1:J] == 1 | CR.trt[,1:J] == 3)
  # assign treatment 2 (Text message) to those clinics who were
  # randomized to conditions 2 or 3
  trt2 <- as.numeric(CR.trt[,1:J] == 2 | CR.trt[,1:J] == 3)
  # add total balance score in the output 
  total.bal <- CR.trt[,J+1]
  CR <- cbind(data, trt1, trt2, total.bal) 
  return(CR)
}

## Appendix B Simulation results controlling for cluster-level covariates

**Table 7:** Simulation results for the effect of treatment with 8 clusters, based on an analysis model that controls for cluster-level covariates

Covariate SD	Degree Confounding	%Bias	Var	MSE	Cov	Power	CI Width	Type 1 Error	Balance

*Covariate Constrained Randomization*									
	*β* = 0.0	−0.19	2.00	2.00	0.93	0.54	10.16	0.07	2.19
*σ_x_* = 0.5	*β* = 0.5	−0.19	2.00	2.00	0.93	0.54	10.16	0.07	2.19
	*β* = 1.0	−0.19	2.00	2.00	0.93	0.54	10.16	0.07	2.19

	*β* = 0.0	−0.19	2.00	2.00	0.93	0.54	10.17	0.07	2.19
*σ_x_* = 1	*β* = 0.5	−0.19	2.00	2.00	0.93	0.54	10.17	0.07	2.19
	*β* = 1.0	−0.19	2.00	2.00	0.93	0.54	10.17	0.07	2.19

	*β* = 0.0	−0.20	2.00	2.00	0.93	0.54	10.17	0.07	2.19
*σ_x_* = 2	*β* = 0.5	−0.20	2.00	2.00	0.93	0.54	10.17	0.07	2.19
	*β* = 1.0	−0.20	2.00	2.00	0.93	0.54	10.17	0.07	2.19

*Simple Randomization*									
	*β* = 0.0	0.10	3.95	3.95	0.93	0.43	13.35	0.07	4.50
*σ_x_* = 0.5	*β* = 0.5	0.10	3.95	3.95	0.93	0.43	13.35	0.07	4.50
	*β* = 1.0	0.10	3.95	3.95	0.93	0.43	13.35	0.07	4.50

	*β* = 0.0	0.10	3.95	3.95	0.93	0.43	13.35	0.07	4.50
*σ_x_* = 1	*β* = 0.5	0.10	3.95	3.95	0.93	0.43	13.35	0.07	4.50
	*β* = 1.0	0.10	3.95	3.95	0.93	0.43	13.35	0.07	4.50

	*β* = 0.0	0.10	3.95	3.95	0.93	0.43	13.35	0.07	4.50
*σ_x_* = 2	*β* = 0.5	0.10	3.95	3.95	0.93	0.43	13.35	0.07	4.50
	*β* = 1.0	0.10	3.95	3.95	0.93	0.43	13.35	0.07	4.50

**Table 8:** Simulation results for the effect of treatment with 12 clusters, based on an analysis model that controls for cluster-level covariates

Covariate SD	Degree Confounding	%Bias	Var	MSE	Cov	Power	CI Width	Type 1 Error	Balance

*Covariate Constrained Randomization*									
	*β* = 0.0	−0.39	0.89	0.89	0.95	0.99	4.42	0.05	1.27
*σ_x_* = 0.5	*β* = 0.5	−0.39	0.89	0.89	0.95	0.99	4.42	0.05	1.27
	*β* = 1.0	−0.39	0.89	0.89	0.95	0.99	4.42	0.05	1.27

	*β* = 0.0	−0.39	0.89	0.89	0.95	0.99	4.42	0.05	1.27
*σ_x_* = 1	*β* = 0.5	−0.39	0.89	0.89	0.95	0.99	4.42	0.05	1.27
	*β* = 1.0	−0.39	0.89	0.89	0.95	0.99	4.42	0.05	1.27

	*β* = 0.0	−0.39	0.89	0.89	0.95	0.99	4.42	0.05	1.27
*σ_x_* = 2	*β* = 0.5	−0.39	0.89	0.89	0.95	0.99	4.42	0.05	1.27
	*β* = 1.0	−0.39	0.89	0.89	0.95	0.99	4.42	0.05	1.27

*Simple Randomization*									
	*β* = 0.0	−0.04	1.19	1.19	0.95	0.95	5.03	0.05	2.98
*σ_x_* = 0.5	*β* = 0.5	−0.04	1.19	1.19	0.95	0.95	5.03	0.05	2.98
	*β* = 1.0	−0.04	1.19	1.19	0.95	0.95	5.03	0.05	2.98

	*β* = 0.0	−0.04	1.19	1.19	0.95	0.95	5.03	0.05	2.98
*σ_x_* = 1	*β* = 0.5	−0.04	1.19	1.19	0.95	0.95	5.03	0.05	2.98
	*β* = 1.0	−0.04	1.19	1.19	0.95	0.95	5.03	0.05	2.98

	*β* = 0.0	−0.04	1.19	1.19	0.95	0.95	5.03	0.05	2.98
*σ_x_* = 2	*β* = 0.5	−0.04	1.19	1.19	0.95	0.95	5.03	0.05	2.98
	*β* = 1.0	−0.04	1.19	1.19	0.95	0.95	5.03	0.05	2.98

## List of abbreviations

CRT: Cluster randomized trial
CR: Covariate-constrained randomization
BEGIN: Behavioral Nudges for Diabetes Prevention study
T2D: Type 2 diabetes
ICC: intra-cluster correlation
MSE: mean squared error

## References

[1] Murray D.M., Taljaard M., Turner E.L., George S.M.. Essential ingredients and innovations in the design and analysis of group-randomized trials. Annual Review of Public Health 2020;41(1):1–19. 10.1146/annurev-publhealth-040119-094027 31869281

[2] Giraudeau B., Ravaud P.. Preventing bias in cluster randomised trials. PLoS Med 2009;6(5):e1000065. 19536323 10.1371/journal.pmed.1000065 PMC2668175

[3] Ivers N.M., Halperin I.J., Barnsley J., Grimshaw J.M., Shah B.R., Tu K., Upshur R., Zwarenstein M.. Allocation techniques for balance at baseline in cluster randomized trials: a methodological review. Trials 2012;13(1):1–9. 22853820 10.1186/1745-6215-13-120 PMC3503622

[4] Dziak J.J., Nahum-Shani I., Collins L.M.. Multilevel factorial experiments for developing behavioral interventions: power, sample size, and resource considerations. Psychological Methods 2012;17(2):153. 22309956 10.1037/a0026972 PMC3351535

[5] Moerbeek M., van Schie S.. How large are the consequences of covariate imbalance in cluster randomized trials: a simulation study with a continuous outcome and a binary covariate at the cluster level. BMC Medical Research Methodology 2016;16(1):1–10. 27401771 10.1186/s12874-016-0182-7 PMC4939594

[6] Li F., Lokhnygina Y., Murray D.M., Heagerty P.J., DeLong E.R.. An evaluation of constrained randomization for the design and analysis of group-randomized trials. Statistics in Medicine 2016;35(10):1565–1579. 26598212 10.1002/sim.6813 PMC4826850

[7] Raab G.M., Butcher I.. Balance in cluster randomized trials. Statistics in Medicine 2001;20(3):351–365. 11180306 10.1002/1097-0258(20010215)20:3<351::aid-sim797>3.0.co;2-c

[8] Yu H., Li F., Gallis J.A., Turner E.L.. cvcrand: A package for covariate-constrained randomization and the clustered permutation test for cluster randomized trials. R Journal 2019;9(2).

[9] Zhou Y., Turner E.L., Simmons R.A., Li F.. Constrained randomization and statistical inference for multi-arm parallel cluster randomized controlled trials. Statistics in Medicine 2022;41(10):1862–1883. 35146788 10.1002/sim.9333 PMC9007899

[10] Watson S.I., Girling A., Hemming K.. Design and analysis of three-arm parallel cluster randomized trials with small numbers of clusters. Statistics in Medicine 2021;40(5):1133–1146. 33258219 10.1002/sim.8828

[11] Ciolino J.D., Diebold A., Jensen J.K., Rouleau G.W., Koloms K.K., Tandon D.. Choosing an imbalance metric for covariate-constrained randomization in multiple-arm cluster-randomized trials. Trials 2019;20(1):1–10. 31138319 10.1186/s13063-019-3324-5 PMC6537428

[12] Vargas M.C., Pineda G.J., Talamantes V., Toledo M.J.L., Owen A., Carcamo P., Gibbert W., Ackermann R.T., Kandula N.R., Cameron K.A., Siddique J., Williams G.C., O’Brien M.J.. Design and rationale of behavioral nudges for diabetes prevention (BEGIN): A pragmatic, cluster randomized trial of text messaging and a decision aid intervention for primary care patients with prediabetes. Contemporary Clinical Trials 2023;:107216. 10.1016/j.cct.2023.107216 PMC10330561 37169219

[13] Menke A., Casagrande S., Geiss L., Cowie C.C.. Prevalence of and trends in diabetes among adults in the united states, 1988–2012. JAMA 2015;314(10):1021–1029. 26348752 10.1001/jama.2015.10029

[14] Knowler W.C., Barrett-Connor E., Fowler S.E., Hamman R.F., Lachin J.M., Walker E.A., Nathan D.M., Watson P., Mendoza J., Smith K., Reduction in the incidence of type 2 diabetes with lifestyle intervention or metformin. New England Journal of Medicine 2002;346(6):393–403. 11832527 10.1056/NEJMoa012512 PMC1370926

[15] Hing E., Uddin S.. Visits to primary care delivery sites: United States, 2008. 47; US Department of Health and Human Services, Centers for Disease Control and Prevention; 2010.

[16] Kalish L.A., Begg C.B.. Treatment allocation methods in clinical trials: a review. Statistics in Medicine 1985;4(2):129–144. 3895341 10.1002/sim.4780040204

